# Prognostic impact of MGMT promoter methylation and MGMT and CD133 expression in colorectal adenocarcinoma

**DOI:** 10.1186/1471-2407-14-511

**Published:** 2014-07-11

**Authors:** Jaime Antonio Oliver, Raúl Ortiz, Consolación Melguizo, Pablo Juan Álvarez, Jaime Gómez-Millán, Jose Prados

**Affiliations:** 1 Institute of Biopathology and Regenerative Medicine (IBIMER), University of Granada, Granada 18100, Spain; 2 Department of Health Sciences, University of Jaén, Jaén 23071, Spain; 3 Biosanitary Institute of Granada (ibs.GRANADA), SAS-Universidad de Granada, Granada, Spain; 4 Department of Anatomy and Embryology, University of Granada, Granada 18012, Spain; 5 Radiation Oncology Department, Hospital Clinico Universitario Virgen de la Victoria, Málaga 29010, Spain

**Keywords:** Colorectal cancer, MGMT, CD133, Methylation status, Biomarker, Overall survival, Disease free-survival

## Abstract

**Background:**

New biomarkers are needed for the prognosis of advanced colorectal cancer, which remains incurable by conventional treatments. O^6^-methylguanine DNA methyltransferase (MGMT) methylation and protein expression have been related to colorectal cancer treatment failure and tumor progression. Moreover, the presence in these tumors of cancer stem cells, which are characterized by CD133 expression, has been associated with chemoresistance, radioresistance, metastasis, and local recurrence. The objective of this study was to determine the prognostic value of CD133 and MGMT and their possible interaction in colorectal cancer patients.

**Methods:**

MGMT and CD133 expression was analyzed by immunohistochemistry in 123 paraffin-embedded colorectal adenocarcinoma samples, obtaining the percentage staining and intensity. MGMT promoter methylation status was obtained by using bisulfite modification and methylation-specific PCR (MSP). These values were correlated with clinical data, including overall survival (OS), disease-free survival (DFS), tumor stage, and differentiation grade.

**Results:**

Low MGMT expression intensity was significantly correlated with shorter OS and was a prognostic factor independently of treatment and histopathological variables. High percentage of CD133 expression was significantly correlated with shorter DFS but was not an independent factor. Patients with low-intensity MGMT expression and ≥50% CD133 expression had the poorest DFS and OS outcomes.

**Conclusions:**

Our results support the hypothesis that MGMT expression may be an OS biomarker as useful as tumor stage or differentiation grade and that CD133 expression may be a predictive biomarker of DFS. Thus, MGMT and CD133 may both be useful for determining the prognosis of colorectal cancer patients and to identify those requiring more aggressive adjuvant therapies. Future studies will be necessary to determine its clinical utility.

## Background

According to the World Health Organization (WHO), colorectal cancer (CRC) is the third most common cancer in males and the second in females and is the fourth cause of cancer death. The WHO expects an increase in CRC incidence and mortality, with estimates of around 1,471,808 newly diagnosed patients and 726,028 deaths worldwide in 2015 [1]. Almost all (95%) of these new CRCs are likely to be adenocarcinomas and, despite recent advances in detection and therapy, 25% of these patients will develop metastasis and have a very low 5-year survival rate of around 10% [2,3]. New biomarkers of CRC are needed to permit an earlier diagnosis and to predict the response to treatment.

Screening for the early detection of CRC is the most effective approach against this disease [4]. Carcinoembryonic antigen (CEA) is recommended as a biomarker to detect spread of the cancer and to follow up CRC patients. However, in the diagnosis of early CRC it has major limitations such as low sensitivity and specificity (36% and 87% respectively). In addition, until a rate of 16% may be false positives [5,6]. Novel biomarkers such as O^6^-methylguanine-DNA methyltransferase (MGMT) and CD133 have been proposed as useful tools for the diagnosis, prognosis, and follow-up of CRC and for the detection of relapse [7]. MGMT is a DNA repair protein that removes O^6^-guanine adducts from DNA [8]. MGMT restores mutagenic O^6^-methylguanine to guanine in normal colonic tissue, preventing DNA alkylation damage [9]. MGMT hypermethylation in CpG islands and low MGMT protein expression appear to be early events in CRC patients. This MGMT epigenetic silencing may lead to G:C to A:T transition mutations in *p53*[10], *K-ras*[11-13], *PIK3CA*[11,14], and *hMLH1*[15], among others. Furthermore, CD133, a transmembrane glycoprotein related to cell-cell interaction and signal transduction, has been associated with cancer stem cells (CSCs), including those in CRC [16]. This CSC subpopulation represents a small number of tumor cells that can self-renew indefinitely and recreate parent tumor cells expressing different surface biomarkers [17]. This marker permits the hierarchical organization of tumor heterogeneity, dividing CRC cells between CD133-positive (CSCs) and CD133-negative cells (non-CSCs) cells [18]. CD133-positive CRC cells have shown special properties, including the capacity to form tumors in xenografts [19], chemo- and radioresistance [20,21], and metastasis promotion [22,23]. Previous studies associated CSC chemo/radio-resistance to MGMT expression in other cancers [24-26].

The aim of the present study was to analyze the clinical implications of MGMT and CD133 in CRC and the possible interactions between them in order to develop a new prognostic biomarker for these patients. Immunohistochemical analysis of MGMT and CD133 expression was carried out in colorectal cancer samples from 123 patients, and MGMT methylation status was determined by methylation-specific PCR (MSP). The expression pattern of the two molecules and MGMT methylation status were correlated with overall survival (OS), disease-free survival (DFS), tumor stage, and differentiation grade, among others. MGMT expression intensity and percentage CD133 expression may be clinically useful for CRC prognosis, but this does not appear to be the case for MGMT methylation status or CD133 expression intensity.

## Methods

### Clinical tissue samples

In this cross-sectional study (case-series design), colorrectal adenocarcinoma samples were obtained from patients at three hospitals in Southern Spain (Puerta del Mar Hospital, Cádiz; Puerto Real Hospital, Cádiz; and San Cecilio Hospital, Granada) between 2004 and 2009. Clinical data of the patients were obtained from the hospital records. Written informed consent was obtained from all patients and controls before their enrolment in the study. The study protocol was approved by the Biomedical Investigation Ethic Committee (Consejeria de Salud; Servicio Andaluz de Salud). Paraffin-embedded tumor specimens were obtained from 123 CRC patients. The differentiation grade and tumor stage were determined according to standard histopathological criteria by two expert pathologists [27]. DNA extraction and analysis, MGMT methylation status test, tissue microarray (TMA) construction, and MGMT and CD133 immunohistochemical analyses were performed in samples from each specimen. None of the patients had received any pre-operative treatment. After the tumor resection, most patients had been treated with chemotherapy (5-fluorouracil [5-FU], oxaliplatin, and/or irinotecan) and/or radiotherapy according to their clinical characteristics.

### DNA extraction, bisulfite treatment, and methylation-specific PCR

DNA was extracted from waxed tissue samples by using the Chemagic MSM I robot (Chemagen, Germany, Baesweiler) in accordance with the manufacturer’s standard recommendations. Determination of methylation patterns in MGMT promoter CpG islands was based on the chemical modification of unmethylated (but not methylated) cytosine to uracil. MSP was performed with specific primers for either methylated or unmethylated DNA, as previously described [10]. Briefly, a 2-μg DNA sample was denatured with sodium hydroxide, modified with sodium bisulfite, and then purified (EpiTect Bisulfite kit, Qiagen, USA, Maryland). Primer sequences were 5′-TTTGTGTTTTGATGTTTGTAGGTTTTTGT-3′ (forward primer) and 5′-AACTCCACACTCTTCCAAAAACAAAACA-3′ (reverse primer) for the unmethylated (UM) reaction and were 5′-TTTCGACGTTCTAGGTTTTCGC-3′ (forward primer) and 5′-GCACTCTTCCGAAAACGAAACG-3′ (reverse primer) for the methylated (M) reaction. PCR-amplified products were electrophoresed on 3% agarose gels, visualized by staining with ethidium bromide, and examined under UV illumination. A sample was classified as hypermethylated when the methylation amplification product alone was observed, partially methylated when both methylated and unmethylated amplification products were seen, and unmethylated when it showed unmethylated amplification products alone. For the statistical analysis, the hypermethylated and partially methylated samples were considered as the methylated (M) group and compared with the unmethylated (UM) group.

### Immunohistochemistry

Formalin-fixed paraffin-embedded CRC tumor samples were used in the construction of TMAs. Briefly, four representative areas were selected from whole hematoxylin-eosin tissue sections of each adenocarcinoma specimen. Cores with diameter of 1 mm were placed 0.8 mm apart in a grid layout using a Manual Tissue Microarrayer (Beecher Instruments, Silver Spring, MD). The resulting tissue microarray blocks were cut into 5-μm sections with a microtome, placed on slides by the adhesive tape-transfer method (Instrumedics, Inc., Hackensack, NJ), and UV cross-linked. TMA dewaxing, rehydration, epitope recovery, and all staining procedures were performed at the same time with the DakoAutostainer EnVision™ FLEX kit (Dako, Barcelona, Spain) using antibodies against MGMT (1:50, Santa Cruz Biotechnology, Inc., Heidelberg, Germany) and CD133 (1:50, Miltenyi Biotec, Bergisch Gladbach, Germany). The antibodies were incubated with *3.3′-*diaminobenzidine (DAB) substrate-chromogen, resulting in a brown-colored precipitate at the antigen site, and cell nuclei were visualized with hematoxylin (blue) counterstaining; nerve tissue was used as a positive control [28]. The readings were done by two experienced pathologists under light microscopy. In the most of specimens, there weren’t significant differences between observers and sample punches. Furthermore, the patients with heterogeneous staining for any antibody were not included in this study. The MGMT staining of tumor cells was scored and grouped as low expression (<50%) (-, +, and ++ scores) and high expression (≥50%) (+++ and ++++ scores). CD133 staining of tumor glands was classified as 0%, <50%, or ≥50%; CD133 staining on the apical and/or endoluminal surface of tumor glands and/or on cell debris was considered positive, in accordance with previous studies [29]. For the statistical analysis, CD133 expression was considered in two categories: low (<50%) or high (≥50%). The intensity of MGMT and CD133 staining was scored as low or high. Thereby, percentage only consider number of cells or tumor glands stained for MGMT and CD133 independently to dye distribution; moreover, intensity was scored high when nucleus or tumor glands were completely stained and low when showed partially or lack of staining regardless of number of cells or tumor glands stained.

### Statistical analysis

Contingency tables and associations were analyzed with the chi-square (χ^2^) test and Fisher’s exact test. DFS (time elapsed between diagnosis and disease recurrence) and OS (time between diagnosis and death) curves were estimated with the Kaplan-Meier method. A two-sided log-rank test was used to determine significant differences between independent curves and patient groups. Significant prognostic factors associated with DFS and/or OS were identified by applying the Cox proportional hazards model, which was constructed using the most relevant molecular, histopathological, and treatment variables. SPSS version 15.0 was used for the data analyses; p < 0.05 was considered significant.

## Results

### Patient characteristics

Table 1 summarizes the characteristics of the 123 patients in the study (65% males, 35% females); the mean (± standard deviation) age was 71.73 ± 10.57 yrs (range, 40 to 93 yrs). The tumor was in stage III in 40.2% (49/122) of patients and the differentiation grade was moderate in 50.4% (59/117). At the most recent follow up, 17.8% (18/101) of the patients had died due to the colorectal adenocarcinoma and 41.9% (44/105) of the patients did not respond to treatment, evidencing local or distal recurrence. The follow-up period ranged between 2 and 93 months. The mean OS of the whole sample was 40.20 ± 22.09 months and the mean DFS was 35.98 ± 24.75 months.

**Table 1:** Clinical characteristics of colon adenocarcinoma patients

**Feature**	**Classification**	**n (%)**
**Sex**	Male	80 (65)
	Female	43 (35)
**Age**	≥50 years	116 (94.3)
	<50 years	7 (5.7)
**Tumor differentiation grade**	Well differentiated	37 (31.6)
	Moderately differentiated	59 (50.4)
	Poorly differentiated	21 (17.9)
**Tumor stage**	I	14 (11.5)
	II	44 (36.1)
	III	49 (40.2)
	IV	15 (12.3)
**Radiotherapy treatment**	Did not receive radiotherapy	96 (87.3)
	Received radiotherapy	14 (12.7)
**Chemotherapy treatment**	Did not receive chemotherapy	47 (38.8)
	Received chemotherapy	74 (61.2)
**Treatment**	No chemotherapy or radiotherapy	40 (36.4)
	Some treatment	70 (63.6)
**Treatment response**	Response	61 (58.1)
	No response	44 (41.9)
**Last follow-up status**	Alive without disease	66 (65.3)
	Alive with disease	17 (16.8)
	Disease progression and death	18 (17.8)

Sample size for sex and age (n = 123), for tumor differentiation grade (n = 117), for tumor stage (n = 122), for radiotherapy treatment (n = 110), for chemotherapy treatment (n = 121), for a treatment (n = 110), for treatment response (n = 105) and last follow-up status (n = 101).

### MGMT promoter methylation status and MGMT expression

**Figure 1:**
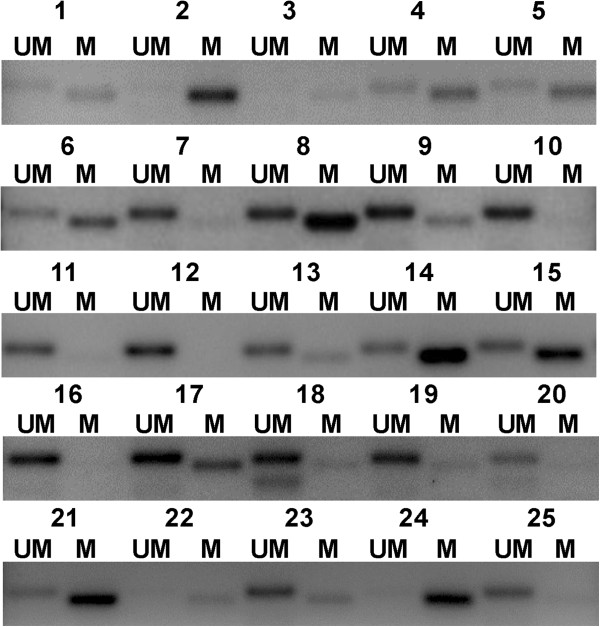
**Methylation-specific PCR analysis of the MGMT promoter in colorectal adenocarcinoma tissue samples.** Representative image showing MGMT promoter methylation study of 25 of the 110 analyzed patients. Partially-methylated patients showed amplification of both UM and M lanes. Hypermethylated patients showed only amplification of M lane. Unmethylated (UM) patients showed lack of M lane.

Table 2 summarizes the molecular characteristics of the patients. MGMT promoter methylation status could be determined in 89.4% (110/123) of the tumors; PCR amplification was unsuccessful or evaluation was not possible in the remaining 13 specimens. The M group included 78.2% of the 110 cases and the UM group 21.8%. Out of the M group, 75.6% showed partial methylation (amplification with UM and M primers) and 24.4% hypermethylation (amplification with M primer alone) (Figure 1).

**Table 2:** Molecular characteristics of colon adenocarcinoma patients

**Feature**	**Classification**	**n (%)**
***MGMT *** **methylation status**	Unmethylated	24 (21.8)
	Methylated	86 (78.2)
**Percentage MGMT expression**	Low	55 (48.2)
	High	59 (51.8)
**MGMT expression intensity**	Low	30 (26.3)
	High	84 (73.7)
**Percentage CD133 expression**	Low	52 (47.3)
	High	58 (52.7)
**CD133 expression intensity**	Low	70 (63.6)
	High	40 (36.4)

Sample size for MGMT methylation status (n = 110), for percentage MGMT expression (n = 114), MGMT expression intensity (n = 114), percentage CD133 expression (n = 110), and CD133 expression intensity (n = 110).

**Figure 2:**
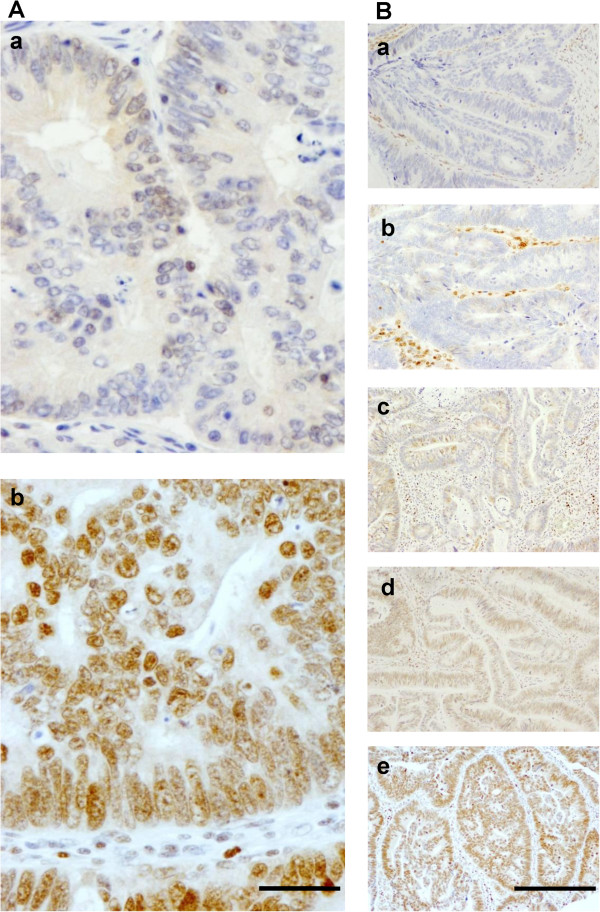
**Immunohistochemical MGMT staining in colorectal adenocarcinoma tissue samples. (A)** Representative photomicrographs of TMA punches illustrating low (a) and high (b) MGMT expression intensity; bar, 50 μm. **(B)** Photomicrographs of TMA punches illustrating different percentages MGMT expression levels: negative (a), <50% (b and c) and ≥50% (d and e); bar, 200 μm.

MGMT staining was always nuclear in colorectal adenocarcinoma gland cells and always detected in surrounding tissue (Figure 2). Data on the percentage MGMT expression were available for 92.7% (114/123) of the patients. Out of these 114 cases, no expression was observed in 15.8%, and expression was scored as + in 2.6%, ++ in 29.8%, +++ in 43.9%, and ++++ in 7.9% (Figure 2); hence, low (<50%) MGMT expression was observed in 48.2% of cases and high expression (≥50%) in 51.8%. No association was found between percentage MGMT expression and MGMT promoter methylation status (Additional file 1: Table S1). A low intensity of MGMT expression was observed in 26.3% of the 114 patients and a high intensity in 73.7% (Figure 2). A significant association was found between low MGMT expression intensity and MGMT promoter methylated (M) (Additional file 1: Table S1). An association was observed between poor-tumor differentiation grade and methylated MGMT promoter (M) (Additional file 2: Table S2).

### Influence of MGMT promoter methylation status and MGMT protein expression on overall survival and disease-free survival

**Figure 3:**
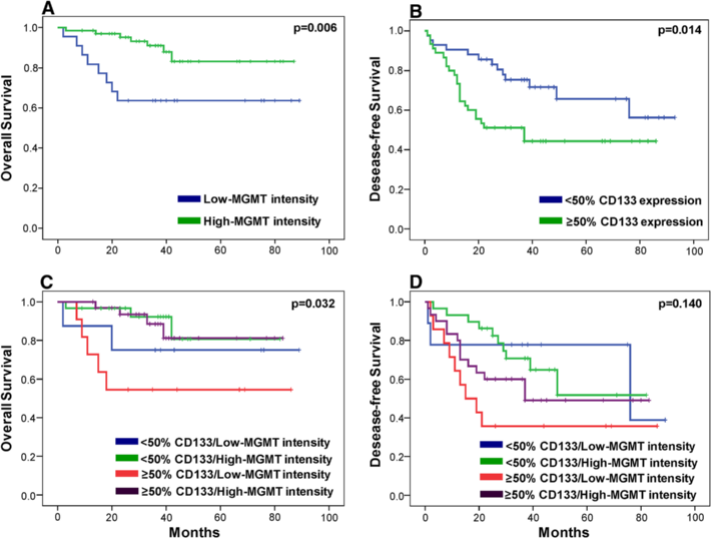
**Kaplan-Meier survival curves according to MGMT and/or CD133 expression in colorectal adenocarcinoma patients. (A)** OS curves for different MGMT expression intensity scores. **(B)** DFS curves for different percentage CD133 expression scores. **(C)** OS curves for different MGMT and CD133 scores when analyzed together and **(D)** DFS curves for different MGMT and CD133 scores when analyzed together.

The mean OS was 61.36 months in patients with low-intensity MGMT expression *versus* 77.48 months in those with high-intensity MGMT expression (Table 3); the correlation between OS and MGMT expression intensity was significant (p < 0.01) (Figure 3). MGMT expression intensity was a prognostic factor for OS after adjusting for treatment and histopathology variables (Table 4). No significant correlation was found between OS and MGMT promoter methylation status or percentage MGMT expression (Table 3). No significant correlation was observed between DFS and MGMT methylation status, MGMT expression intensity, or percentage MGMT expression (Table 3).

**Table 3:** Interaction of overall survival (OS) and disease-free survival (DFS) with histopathological variables

		**OS**		**DFS**	
**Variables**		**Mean (95% CI)**	**p value**	**Mean (95% CI)**	**p value**
**Sex**	Male	72.08 (62.61-81.55)	0.103	52.15 (42.12-62.18)	0.179
	Female	79.52 (71.55-87.50)		61.00 (49.16-72.85)	
**Differentiation grade**	Well-moderate	77.62 (70.03-85.21)	0.408	56.25 (47.45-65.06)	0.649
	Poor	64.14 (48.53-79.75)		56.50 (38.35-74.64)	
**Tumor stage**	I-II	80.75 (71.71-89.78)	0.167	70.31 (59.84-80.78)	0.002*
	III-IV	68.89 (58.72-79.05)		42.44 (32.30-52.58)	
***MGMT *** **methylation status**	Unmethylated	73.54 (65.17-81.90)	0.398	49.87 (34.54-65.20)	0.949
	Methylated	76.07 (67.82-84.32)		57.33 (47.55-67.12)	
**Percentage MGMT expression**	Low	70.53 (60.02-81.05)	0.211	50.77 (39.35-62.20)	0.328
	High	77.04 (69.58-84.50)		58.62 (48.37-68.87)	
**MGMT expression intensity**	Low	61.36 (45.99-76.72)	0.006*	47.76 (32.80-62.71)	0.171
	High	77.48 (70.75-84.21)		56.43 (47.39-65.47)	
**Percentage CD133 expression**	Low	82.03 (72.97-91.10)	0.273	67.91 (56.68-79.14)	0.014*
	High	70.41 (61.33-79.50)		46.01 (35.06-56.97)	
**CD133 expression intensity**	Low	77.90 (69.39-86.41)	0.642	59.76 (49.62-69.89)	0.517
	High	78.00 (67.92-88.08)		53.74 (39.44-68.05)	
**High MGMT intensity**	CD133 ≥ 50%	73.06 (63.99-82.14)	0.032*	49.14 (36.25-62.04)	0.140
	CD133 < 50%	72.33 (61.82-82.84)		57.08 (44.64-69.51)	
**Low MGMT intensity**	CD133 ≥ 50%	52.36 (30.53-74.19)		37.85 (18.87-56.83)	
	CD133 < 50%	69.50 (45.88-93.11)		64.50 (41.41-87.59)	

Statistically significant variables (*p < 0.05). CI, confidence interval.

**Table 4:** Multivariate analysis: cox proportional hazards model for OS and DFS

	**OS**		**DFS**	
**Variables**	**HR (CI)**	**p value**	**HR (CI)**	**p value**
**Sex (male/female)**	2.69 (0.77-9.34)	0.118	1.56 (0.80-3.05)	0.186
**Differentiation grade (well-moderate/poor)**	0.62 (0.20-1.93)	0.412	1.24 (0.48-3.17)	0.652
**Tumor grade (I-II/III-VI)**	0.50 (0.18-1.35)	0.175	0.38 (0.19-0.73)	0.004*
**Radiotherapy (no/yes)**	0.41 (0.13-1.27)	0.123	0.64 (0.29-1.41)	0.276
**Chemotherapy (no/yes)**	0.48 (0.16-1.49)	0.210	0.50 (0.25-0.99)	0.049*
**MGMT expression intensity (low/high)**	3.73 (1.35-10.33)	0.011*	1.55 (0.81-2.99)	0.182
**Percentage CD133 expression (low/high)**	0.54 (0.18-1.65)	0.280	0.44 (0.22-0.86)	0.018*

HR, Hazard ratio. CI, confidence interval. Statistically significant variables (*p < 0.05).

### CD133 protein expression

**Figure 4:**
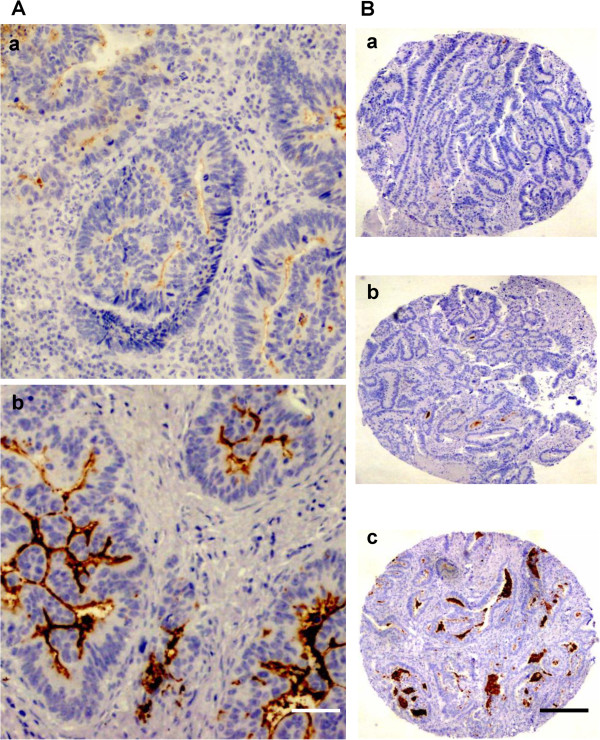
**Immunohistochemical CD133 staining in colorectal adenocarcinoma tissue samples. (A)** Representative photomicrographs of TMA punches illustrating low (a) and high (b) CD133 expression intensity; bar, 50 μm. **(B)** Photomicrographs of TMA punches illustrating different percentage CD133 expression levels: negative (a), <50% (b), ≥50% (c); bar, 200 μm.

CD133 expression results were available for 110 (89.4%) of the patients (Table 2). CD133 expression was detected on the endoluminal surface of tumor glands and on cell debris; no staining was observed in other tumor regions or in normal tissues. Out of the 110 specimens analyzed, the staining was scored as 0% in 12.7%, <50% in 34.6%, and ≥50% in 52.7% (Figure 4). In addition, 36.4% of the 110 patients had a high-intensity CD133 expression and 63.6% a low-intensity expression (Figure 4). An association was observed between no treatment response and high CD133 percentage expression (Additional file 3: Table S3).

### Influence of CD133 protein expression on overall survival and disease-free survival

OS was not significantly associated with CD133 protein expression intensity or percentage (Table 3). However, a statistically significant correlation (p < 0.05) was observed between DFS and percentage CD133 expression (Figure 3), with a mean DFS of 67.91 months in those with low (<50%) expression *versus* 46.01 months in those with higher (≥50%) CD133 expression (Table 3). The tumor stage was also correlated with DFS (Table 3). The multivariable analysis results showed that CD133 protein expression was not an independent prognostic factor (Table 4).

### MGMT and CD133 interactions and clinical influence

No significant association was found between MGMT promoter methylation or MGMT expression percentage/intensity and CD133 expression percentage/intensity (Additional file 1: Table S1). CD133 expression percentage and MGMT intensity could be compared in 109 patients. Among the patients with low MGMT intensity, CD133 expression percentage was low in 42.9% and high in 57.1%. Among the patients with high MGMT intensity, CD133 expression percentage was low in 48.1% and high in 51.9%.

Study of the potential relationship of MGMT intensity and percentage CD133 expression with clinical outcome variables (Figure 3) revealed a significant correlation with OS but no significant correlation with DFS (Table 3). The patients with low-intensity MGMT expression and high (≥50%) CD133 expression had the worst OS (52.36 months) and DFS (37.85 months) outcomes (Table 3).

## Discussion

In this study of tumors from CRC patients, methylated MGMT promoter was significantly associated with low MGMT expression intensity and poor-differentiation grade but not with OS, DFS, or tumor stage. High MGMT expression intensity was correlated with longer OS but not with DFS, tumor stage, or differentiation grade. High percentage of CD133 expression was correlated with shorter DFS but not with OS, tumor stage, or differentiation grade. MGMT expression intensity can be considered as an independent prognostic factor for OS, but the influence of percentage CD133 expression on the prognosis for DFS also depends on the tumor stage.

The relevance of MGMT in CRC carcinogenesis is widely accepted, and reduced MGMT expression has been documented in tumor *versus* normal colon tissue [30]; however, the mechanism by which MGMT expression is controlled remains controversial. Lee *et al.*[31] observed hypermethylated genes, including MGMT, in early stages of colorectal adenoma, and MGMT promoter methylation has been implicated in colon cancer progression (in the adenoma-carcinoma sequence) [31,32]. Sinha *et al.*[33] demonstrated that MGMT promoter methylation was associated with tumor stage, metastasis, and lymphatic invasion in advanced CRC. Various authors have reported the effects of MGMT inactivation on other cancer-related genes. It has been found that the epigenetic silencing of MGMT by promoter hypermethylation can lead to G:C to A:T transition mutations in *p53*[10], *K-ras*[11-13], and *PIK3CA*[11,14], facilitating progression of the tumor to more advanced stages.

Based on the above data, research efforts have focused on the diagnostic and prognostic relevance of MGMT. Various authors have reported that MGMT methylation is a useful marker to detect early stages of CRC [34,35]. Kang *et al*. [36] concluded that a more sensitive screening can be achieved by testing the DNA methylation status of some genes, including MGMT, than by analyzing fecal blood. In addition, Nagasaka *et al*. [37] and Nilsson *et al.*[38] suggested that MGMT hypermethylation in CRC may be related to non-recurrence after chemotherapy and better survival. Experimental data support this possibility, because 5-FU cytotoxicity was enhanced by O6-benzylguanine-induced MGMT depletion in colon cancer cells with high MGMT expression. It was suggested that elevated MGMT levels may be a marker of a low therapeutic response [39], and MGMT hypermethylation was associated with a better prognosis in CRC patients [38]. In contrast, Shima *et al.*[11] found no significant correlation between MGMT promoter status and survival and suggested that this status has little clinical relevance. Our results showed a significant association of methylated MGMT promoter with low-MGMT expression intensity and poor-differentiation grade. However, no correlation was found between MGMT methylation and OS or DFS in CRC patients, whereas high MGMT intensity was correlated with longer OS but not with tumor grade or differentiation. These contradictory results may be related to the multifactorial and complex regulation of MGMT protein expression. Two distinct patterns of MGMT methylation have been associated with different mutations or epigenetic changes in CRC [40], and methylation is not the sole regulatory mechanism of MGMT protein levels [41]. Some MGMT polymorphisms may reduce MGMT activity and/or sensitivity [42,43] and have been associated with progression-free survival in CRC patients [44]. Despite MGMT hypermethylation or lack of MGMT protein has been associated with a better treatment response and survival at short-term [37-39], the epigenetic silencing of MGMT promotes different mutations [10-14] which could facilitate the tumor progression reducing the overall survival at long-term.

CD133 is widely recognized as a stem cell biomarker in normal and cancer colon tissue [45-47]. Its expression was detected in around half of a series of precancerous colon adenomas [48] and was found to be pronounced in invasive margins of colorectal tumors [29]. Other authors reported that CD133 expression is not restricted to intestinal stem or cancer-initiating cells and that both CD133-positive and CD133-negative cells can initiate a tumor [47]. In the present study, the high percentage of CD133 expression was correlated with shorter DFS but not with OS, tumor stage, or differentiation grade in CRC patients*,* suggesting that this molecule may be relevant to determine recurrence. These findings are consistent with the study by Coco *et al.*[49], who found a higher risk of recurrence and death in CRC patients with increased CD133 levels. Reggiani *et al*. [50] concluded that CD133 is useful for the prognosis in stage I CRC patients and for the selection of patients requiring adjuvant treatment. Moreover, Jao *et al.*[51] correlated cytoplasmic CD133 expression with tumor local recurrence and survival in CRC patients. However, a similar study found no correlation between cytoplasmic CD133 and patient survival [52], while Kojima *et al.*[53] observed no differences in DFS between CD133-positive and-negative patients, although they considered CD133 overexpression to be a risk factor in patients with well- and moderately-differentiated adenocarcinomas. CD133 expression on cell debris and the endoluminal surface has also been proposed as CRC biomarker. Horst *et al.*[29] found a significant correlation between endoluminal surface CD133 expression and low survival in CRC patients, while Xi *et al.*[54] reported that CD133 expression in membrane and cytoplasm of cells on the luminal surface of cancerous glands was of prognostic value in CRC patients. All these results are supported by CD133 mRNA studies too. Saigusa *et al.*[55] observed correlation between CD133 mRNA expression and survival and distant recurrence in rectal patients. Further, Kawamoto *et al.*[56] associated recurrence and short DFS with higher CD133 RNAm levels. Similar results observed Yasuda *et al.*[57].

The comparison of results among studies is hampered by methodological differences. Thus, CD133 staining patterns were found to differ in CRC between the use of AC133 (Miltenyi Biotech) and Ab19898 (Abcam) monoclonal antibodies [49] and among the application of anti-CD133 (Cell Signalling), AC133 (Miltenyi Biotech), and polyclonal anti-CD133 (Santa Cruz Biotechnology) antibodies [29]. These staining variations were confirmed in previous glioblastoma studies [58]. In addition, whereas some authors compared cell cytoplasm staining between patients with less and more than 5% CD133 positive cells [49,54,59], others compared the number of CD133-stained glands between patients with less and more than 50% positive glands [29].

Finally, MGMT expression or methylation status has been related to radio-chemo/resistance in the CSC population in some tumors such as glioma [24-26]. He *et al.*[25] reported that patients with methylated MGMT promoter and high CD133 expression had the worst progression-free survival. In contrast, Metellus *et al.*[26] observed shorter OS and progression-free survival in patients with unmethylated MGMT and high CD133 expression. In the present study, no significant association was found between MGMT and CD133 in CRC patients. However, consistent with the findings of He *et al.*[25], DFS and OS outcomes were worse in patients with low MGMT expression intensity and ≥50% CD133 expression.

## Conclusions

Our study evidences the relevance of MGMT and CD133 in the clinical outcome of CRC patients. High MGMT expression intensity was correlated with longer overall survival, while high percentage of CD133 expression was related to shorter-recurrence time lapse. Hence, the intensity of MGMT protein expression and the percentage CD133 protein expression may help to identify patients who need a more aggressive adjuvant therapy.

## Abbreviations

MGMT: O^6^-methylguanine DNA methyltransferase; WHO: World health organization; MSP: Methylation-specific PCR; OS: Overall survival; DFS: Disease-free survival; CRC: Colorectal cancer; CEA: Carcinoembryonic antigen; CSCm: Cancer stem cells; TMA: Tissue microarray; 5-FU: 5-fluorouracil; M: Methylated; UM: Unmethylated; DAB: 3.3′-diaminobenzidine (DAB); HR: Hazard ratio; CI: Confidence interval.

## Competing interests

The authors declare that they have no competing interests.

## Authors’ contributions

Conceived and designed the experiments: CM, JP, RO. Performed the experiments: JAO, RO, CM. Analyzed the data: JAO, RO, CM, PJA, JGM. Wrote the paper: JAO, CM, JP. All authors read and approved the final manusript.

## Pre-publication history

The pre-publication history for this paper can be accessed here:

http://www.biomedcentral.com/1471-2407/14/511/prepub

## Supplementary Material

Additional file 1: Tables S1 Association between molecular variables. Click here for file

Additional file 2: Tables S2 Association between histopathological and MGMT molecular variables. Click here for file

Additional file 3: Tables S3 Association between histopathological variables and CD133 protein expression. Click here for file
