# Supplementary material for: Prognostic impact of MGMT promoter methylation and MGMT and CD133 expression in colorectal adenocarcinoma
Source: BMC Cancer. 2014 Jul 11;14:511. doi: 10.1186/1471-2407-14-511 (PMC4227111; doi:10.1186/1471-2407-14-511)
Supplement: Additional file 1: Tables S1 — Association between molecular variables. [file 1471-2407-14-511-S1.docx]

**Additional file 1: Table S1.**  **Association between molecular variables**

|  |  | **CD133 protein expression** | | | | | | ***MGMT* promoter** | | |
| --- | --- | --- | --- | --- | --- | --- | --- | --- | --- | --- |
|  |  | **Percentage** | | | **Intensity** | | | **Methylation status** | | |
| **Variables** | | Low | High | p value | Low | High | p value | UM | M | p value |
| **Percentage MGMT expression** | Low (%) | 24 (46.2) | 28 (53.8) | 0.899 | 35 (67.3) | 17 (32.7) | 0.407 | 8 (15.4) | 44 (84.6) | 0.135 |
|  | High (%) | 27 (47.4) | 30 (52.6) |  | 34 (59.6) | 23 (40.4) |  | 15 (27.3) | 40 (72.7) |  |
| **MGMT expression intensity** | Low (%) | 12 (42.9) | 16 (57.1) | 0.629 | 21 (75.0) | 7 (25.0) | 0.136 | 0 (00.0) | 28 (100) | 0.001* |
|  | High (%) | 39 (48.1) | 42 (51.9) |  | 48 (59.3) | 33 (40.7) |  | 23 (29.1) | 56 (70.9) |  |
| ***MGMT* methylation status** | UM (%) | 12 (54.5) | 10 (45.5) | 0.494 | 16 (72.7) | 6 (27.3) | 0.360 |  |  |  |
|  | M (%) | 38 (46.3) | 44 (53.7) |  | 51 (62.2) | 31 (37.8) |  |  |  |  |

Statistically significant variables (*p<0.05). UM, unmethylated; M, methylate
