# Supplementary material for: Prognostic impact of MGMT promoter methylation and MGMT and CD133 expression in colorectal adenocarcinoma
Source: BMC Cancer. 2014 Jul 11;14:511. doi: 10.1186/1471-2407-14-511 (PMC4227111; doi:10.1186/1471-2407-14-511)
Supplement: Additional file 2: Tables S2 — Association between histopathological and MGMT molecular variables. [file 1471-2407-14-511-S2.docx]

**Additional file 2: Table S2. Association between histopathological and MGMT molecular variables**

|  |  | ***MGMT*promoter** | | | **MGMT proteinexpression** | | | | | |
| --- | --- | --- | --- | --- | --- | --- | --- | --- | --- | --- |
|  |  | **Methylation status** | | | **Percentage** | | | **Intensity** | | |
| **Variables** | | UM | M | p value | Low | High | p value | Low | High | p value |
| **Sex** | Male (%) | 16 (22.2) | 56 (77.8) | 0.888 | 39 (52.0) | 36 (48.0) | 0.266 | 17 (22.7) | 58 (77.3) | 0.220 |
|  | Female (%) | 8 (21.1) | 30 (78.9) |  | 16 (41.0) | 23 (59.0) |  | 13 (33.3) | 26 (66.7) |  |
| **Age** | <50years (%) | 2 (28.6) | 5 (71.4) | 0.645 | 2 (28.6) | 5 (71.4) | 0.440 | 0 (0.0) | 7 (100) | 0.187 |
|  | ≥50years (%) | 22 (21.4) | 81 (78.6) |  | 53 (49.5) | 54 (50.5) |  | 30 (28.0) | 77 (72.0) |  |
| **Differentiation grade** | Well-moderate (%) | 23 (26.1) | 65 (73.9) | 0.011* | 46 (50.0) | 46 (50.0) | 0.417 | 23 (25.0) | 69 (75.0) | 1 |
|  | Poor (%) | 0 (0.0) | 18 (100) |  | 8 (40.0) | 12 (60.0) |  | 5 (25.0) | 15 (75.0) |  |
| **Tumor stage** | I-II (%) | 13 (24.5) | 40 (75.5) | 0.507 | 26 (47.3) | 29 (52.7) | 0.841 | 15 (27.3) | 40 (72.7) | 0.823 |
|  | III-IV (%) | 11 (19.3) | 46 (80.7) |  | 29 (49.2) | 30 (50.8) |  | 15 (25.4) | 44 (74.6) |  |
| **Treatment response** | No response (%) | 8 (21.6) | 29 (78.4) | 0.683 | 22 (55.0) | 18 (45.0) | 0.211 | 14 (35.0) | 26 (65.0) | 0.127 |
|  | Response (%) | 10 (18.2) | 45 (81.8) |  | 24 (42.1) | 33 (57.9) |  | 12 (21.1) | 45 (78.9) |  |
| **Patient status** | Alive (%) | 16 (21.6) | 58 (78.4) | 0.726 | 32 (41.6) | 45 (58.4) | 0.189 | 14 (18.2) | 63 (81.8) | 0.007* |
|  | Dead (%) | 2 (13.3) | 13 (86.7) |  | 9 (60.0) | 6 (40.0) |  | 8 (52.3) | 7 (46.7) |  |

Statistically significant variables (*p<0.05). UM, unmethylated; M, methylated.
