# Supplementary material for: Prognostic impact of MGMT promoter methylation and MGMT and CD133 expression in colorectal adenocarcinoma
Source: BMC Cancer. 2014 Jul 11;14:511. doi: 10.1186/1471-2407-14-511 (PMC4227111; doi:10.1186/1471-2407-14-511)
Supplement: Additional file 3: Tables S3 — Association between histopathological variables and CD133 protein expression. [file 1471-2407-14-511-S3.docx]

**Additional file 3: Table S3.**  **Association between histopathological variables and CD133 protein expression**

|  |  | **CD133 protein expression** | | | | | |
| --- | --- | --- | --- | --- | --- | --- | --- |
|  |  | **Percentage** | | | **Intensity** | | |
| **Variables** | | Low | High | p value | Low | High | p value |
| **Sex** | Male (%) | 34 (46.6) | 39 (53.4) | 0.837 | 45 (61.6) | 28 (38.4) | 0.542 |
|  | Female (%) | 18 (48.6) | 19 (51.4) |  | 25 (67.6) | 12 (32.4) |  |
| **Age** | <50years (%) | 5 (71.4) | 2 (28.6) | 0.252 | 3 (42.9) | 4 (57.1) | 0.254 |
|  | ≥50years (%) | 47 (45.6) | 56 (54.4) |  | 67 (65.0) | 36 (35.0) |  |
| **Differentiation grade** | Well-moderate (%) | 43 (47.8) | 47 (52.2) | 0.974 | 56 (62.2) | 34 (37.8) | 0.610 |
|  | Poor (%) | 9 (47.4) | 10 (52.6) |  | 13 (68.4) | 6 (31.6) |  |
| **Tumor stage** | I-II (%) | 30 (53.6) | 26 (46.4) | 0.178 | 35 (62.5) | 21 (37.5) | 0.801 |
|  | III-IV (%) | 22 (40.7) | 32 (59.3) |  | 35 (64.8) | 19 (35.2) |  |
| **Treatment response** | No response (%) | 13 (35.1) | 24 (64.9) | 0.046* | 24 (64.9) | 13 (35.1) | 0.966 |
|  | Response (%) | 32 (56.1) | 25 (43.9) |  | 37 (64.9) | 20 (35.1) |  |
| **Patient status** | Alive (%) | 38 (50.0) | 38 (50.0) | 0.325 | 47 (61.8) | 29 (38.2) | 0.494 |
|  | Dead (%) | 5 (36.7) | 9 (64.3) |  | 10 (71.4) | 4 (28.6) |  |

Statistically significant variables (*p<0.05).
